# TSG101 Promotes SIAH1 Auto‐Ubiquitination to Drive Migration and Invasion in Hepatocellular Carcinoma Cells

**DOI:** 10.1111/jcmm.71109

**Published:** 2026-03-23

**Authors:** Jie Qiu, Xixi Gu, Kai Wu, Jinzhao Wu, Chenrui Xu, Wengang Shan, Hengliang Shi

**Affiliations:** ^1^ Center for Sci‐Tech Innovation and Public Service The Affiliated Hospital of Xuzhou Medical University Xuzhou Jiangsu China; ^2^ Institute of Digestive Diseases Xuzhou Medical University Xuzhou Jiangsu China; ^3^ Department of General Surgery The Affiliated Hospital of Xuzhou Medical University Xuzhou Jiangsu China

**Keywords:** hepatocellular carcinoma, SIAH1, TSG101, ubiquitination

## Abstract

Our previous studies have demonstrated that seven in absentia homologue 1 (SIAH1), an E3 ubiquitin ligase, is downregulated in hepatocellular carcinoma (HCC) and regulates substrate ubiquitination. However, the molecular mechanisms governing reduced SIAH1 expression in HCC remain unclear. Here, a yeast two‐hybrid experiment and co‐immunoprecipitation assay identified tumour susceptibility gene 101 (TSG101) as a potential interacting protein of SIAH1. We found that TSG101 negatively regulated the expression of SIAH1. Besides, TSG101 is upregulated in HCC and associated with a poor prognosis. TSG101 promotes the migration and invasion of HCC cells by regulating SIAH1 expression. Molecularly, TSG101 promoted the auto‐ubiquitination and degradation of SIAH1 via the proteasome pathway, thereby reducing its protein stability. Finally, the protein levels of SIAH1 were found to be inversely correlated with TSG101 in human HCC tissues. In summary, TSG101 is up‐regulated in human HCC tissues and promotes the migration and invasion of HCC cells by inducing SIAH1 auto‐ubiquitination and degradation.

**Abbreviations**
CHX: Cycloheximide
HCC: hepatocellular carcinoma
SIAH1: seven in absentia homologue 1
TSG101: tumour susceptibility gene 101

## 1 Introduction

Hepatocellular carcinoma (HCC) is a highly prevalent malignant tumour of the digestive system, characterized by its aggressive behaviour and high metastatic potential [1]. With the rapid advancement of medical therapeutics in recent years, the survival of patients with advanced HCC has been extended using targeted drugs. However, the objective remission rate of drug therapy is still not satisfactory, and some patients with HCC cannot benefit from these treatments [2, 3]. Therefore, it is highly important to explore the mechanisms that contribute to the occurrence and development of HCC and to find new molecular targets that could be utilized for HCC treatment.

SIAH1 (seven in absentia homologue 1) is classified as a RING‐type E3 ubiquitin ligase, exhibiting significant sequence conservation in humans. This protein plays a crucial role in the ubiquitin‐mediated degradation of various substrates in cells [4, 5, 6]. Studies have shown that SIAH1 is related to many cell life activities, such as stress response and gene expression regulation [7, 8]. Moreover, the expression of SIAH1 is closely related to the occurrence and development of breast cancer, gastric cancer, colorectal cancer and ovarian cancer [6, 9, 10, 11]. Our previous research has demonstrated that SIAH1 is down‐regulated in HCC, and can induce the degradation of Cln Three Requiring 9 (CTR9) through ubiquitination, thereby suppressing the metastasis of HCC cells [12]. Besides, we also found that deficiency of SIAH1 promotes the formation of filopodia in liver cancer, which plays an important role in cancer metastasis [13]. Our previous studies primarily focused on how SIAH1 regulates the progression of HCC. However, the factors that regulate SIAH1 itself have not been thoroughly investigated. Therefore, this study aimed to explore the underlying causes of the low expression of SIAH1 in HCC. Auto‐ubiquitination has been reported to occur in many E3 ubiquitin ligases, which play an important role in various regulations of cells. Auto‐ubiquitination of MDM2 enhances its ligase activity [14]. NEDD4‐1 undergoes lysine 29 (K29)‐linked auto‐ubiquitination at K1279, which is essential for autophagy [15]. These E3 ligases usually form a homodimer with themselves and can be ubiquitinated without other E3 ligases. In this study, we focused on SIAH1 auto‐ubiquitination and explored the association with its low expression.

TSG101 (tumour susceptibility gene 101) is a regulatory protein involved in the ubiquitination process, acting as a dominant negative mutant of E2 ubiquitin binding enzyme [16]. It has been reported that TSG101 has multiple functions in cell life activities, such as endosome sorting and transport, transcriptional regulation, cell cycle, cell proliferation, and cell division [17, 18]. In addition, TSG101 is verified to be upregulated in a variety of malignant tumours, including HCC [19, 20, 21, 22]. We have also confirmed that TSG101 is upregulated in human HCC patients, which may accelerate the proliferation, migration, and invasion of HCC cells through regulating paternally expressed gene 10 (PEG10) [23]. These findings indicate that TSG101 may play a significant role in the pathogenesis and progression of HCC. However, the association between TSG101 and SIAH1 remains unclear.

In this study, we found a connection between SIAH1 and TSG101 by yeast two‐hybrid screening and aimed to investigate the interaction and regulatory relationship between them. We gave evidence that TSG101 promoted the migration and invasion of HCC cells by regulating the auto‐ubiquitination and degradation of SIAH1. We also revealed the relevance of TSG101 and SIAH1 expression in clinical samples.

## 2 Materials and Methods

### 2.1 Tissue Samples

The clinical tissue specimens were obtained from the Department of Hepatobiliary and Pancreatic Surgery, Affiliated Hospital of Xuzhou Medical University (Xuzhou, Jiangsu, China), and their origin was confirmed through pathological analysis. This study was approved by the Ethics Review Committee of the Affiliated Hospital of Xuzhou Medical University (approval no.: XYFY2019‐KL129‐01). Informed consent was obtained from all patients before treatment. The study was conducted in accordance with the Declaration of Helsinki.

### 2.2 Cell Culture

The 293 T cell line and the HCC cell lines MHCC97H and Huh7 were provided by the Stem Cell Bank, Chinese Academy of Sciences (Shanghai, China), and were cultured in an incubator at 37°C and 5% CO_2_ with Dulbecco's Modified Eagle Medium (DMEM) (Gibco, Shanghai, China) supplemented with 10% fetal bovine serum (Gibco, Shanghai, China).

### 2.3 Antibodies and Plasmids

Antibodies against His (No. 66005–1‐Ig), mCherry (No. 26765–1‐AP), TSG101 (No. 28283–1‐AP), SIAH1 (No. 13886–1‐AP), MMP2 (No. 10373–2‐AP), MMP9 (No. 10375–2‐AP), Flag (No. 66008–4‐Ig), HA (No. 81290–1‐RR) and β‐actin (No. 66009–1‐Ig) were purchased from Proteintech (Wuhan, Hubei, China). All plasmids were constructed by Youbio (Changsha, Hunan, China).

### 2.4 Yeast Two‐Hybrid Screen

The human SIAH1 cDNA was inserted into the pGBK‐T7 vector to form a bait plasmid. The recombinant pGBK‐T7‐SIAH1 plasmid was transformed into yeast Y2HGold (Clontech, CA). The Y187 yeast containing human cDNA library (Cat No. 630486, Clontech) was hybridized with Y2HGold/pGBK‐T7‐SIAH1 and spread on the SD/−Leu/−Trp/X‐Gal/AbA (Aureobasidin A) (DDO/X/A) agar plate for screening. Then, the blue yeast colonies were patched to the SD/−Ade/His/Leu/Trp/X‐Gal/AbA (QDO/X/A) plate for further confirmation. Subsequently, the survived colonies on the above nutrient‐deficient plate were subjected to plasmid preparation. The isolated plasmids were sequenced using T7 promoter‐specific primer. The inserted gene of the library was determined by basic local alignment search tool (BLAST) analysis of the sequencing results using the NCBI database.

### 2.5 Transwell Migration and Invasion Assays

Transwell migration and invasion assays were carried out as described [12, 23, 24]. Transwell chambers (24‐well, 8.0 μm pore membranes, LABSELECT: #14314, Hefei, Anhui, China) were used in the migration assay. 1 × 10^4^ cells/well were seeded in the upper chamber in 200 μL of serum‐free medium, and 600 μL complete medium as a chemoattractant was added in the lower chamber. After incubated for 24 h at 37°C, some cells successfully passed through the upper chamber membrane, and the cells on the lower surface of the membrane are the migrated cells. After fixed with 4% paraformaldehyde for 30 min and stained with 0.3% crystal violet for 30 min, the migrated cells were photographed by inverted microscope.

The transwell invasion assay was conducted as described above, except that 100 μL of 1 × Matrigel (BD, Shanghai, China) was added to the upper compartment. Three random fields of view in each chamber were selected for counting. The control group was labelled as ‘1’ for statistical purposes.

### 2.6 Western Blotting (WB) Analysis

For western blotting, cells were lysed in RIPA buffer supplemented with a protease inhibitor cocktail and centrifuged at 12,000 × g at 4°C for 10 min; equal amounts of protein were subjected to 10% SDS‐PAGE and then transferred onto a 0.45‐μm pore size PVDF membrane (Merck, Millipore: IPVH00010, Germany). After blocking with 3% bovine serum albumin, the membrane was incubated overnight with the primary antibodies at 4°C and then with secondary antibodies for 1 h at room temperature. After washing with 1 × TBST, ECL Plus western blotting Substrate was used to detect the protein bands, and a chemiluminescence detection system was used for visualization. ImageJ 1.8.0 was used to quantify band density. Relative protein levels were determined by normalizing the optical density values of the target protein with those of the loading control.

### 2.7 Immunoprecipitation (IP) Assay

The 293 T or HCC cells were lysed with ice‐cold IP buffer (1% Triton‐X‐100, 150 mM NaCl, 20 mM HEPES, 2 mM EDTA, 5 mM MgCl_2_, pH 7.4). The cell lysates containing the proteins were conjugated to the beads (MCE: HY‐K0202, USA) after being incubated overnight with the indicated antibodies. Subsequently, the beads were eluted and subjected to western blotting using the indicated primary and corresponding secondary antibodies.

### 2.8 Cycloheximide (CHX) Tracking Assay

CHX (50 μM, MCE: HY‐12320, USA) was used to treat cells silencing or overexpressing TSG101 for a time gradient. The protein levels of SIAH1 and TSG101 or mCherry‐TSG101 were analyzed using western blotting. The decrease of SIAH1 protein level with time represents the decrease of its protein stability.

### 2.9 Statistical Analysis

Data represent the results of experiments repeated at least three times, and all quantitative data are expressed as the mean ± SD. Statistical analysis was performed using GraphPad Prism (v.10.0; GraphPad Software, USA). Student's *t*‐tests were used to compare samples with normality, homogeneity of variance, and independence. Non‐parametric tests were used to analyze measurement or count data that did not meet these requirements. *p* < 0.05 was considered statistically significant.

## 3 Results

### 3.1 SIAH1 Interacts With TSG101 and Is Down‐Regulated by TSG101

Our previous studies have demonstrated that SIAH1 is down‐regulated in HCC, and it acts as an E3 ligase to regulate the ubiquitination and degradation of FASN and CTR9, thereby suppressing the metastasis of HCC cells [12, 13]. However, the underlying causes of the diminished expression of SIAH1 in HCC require further investigation.

**FIGURE 1:**
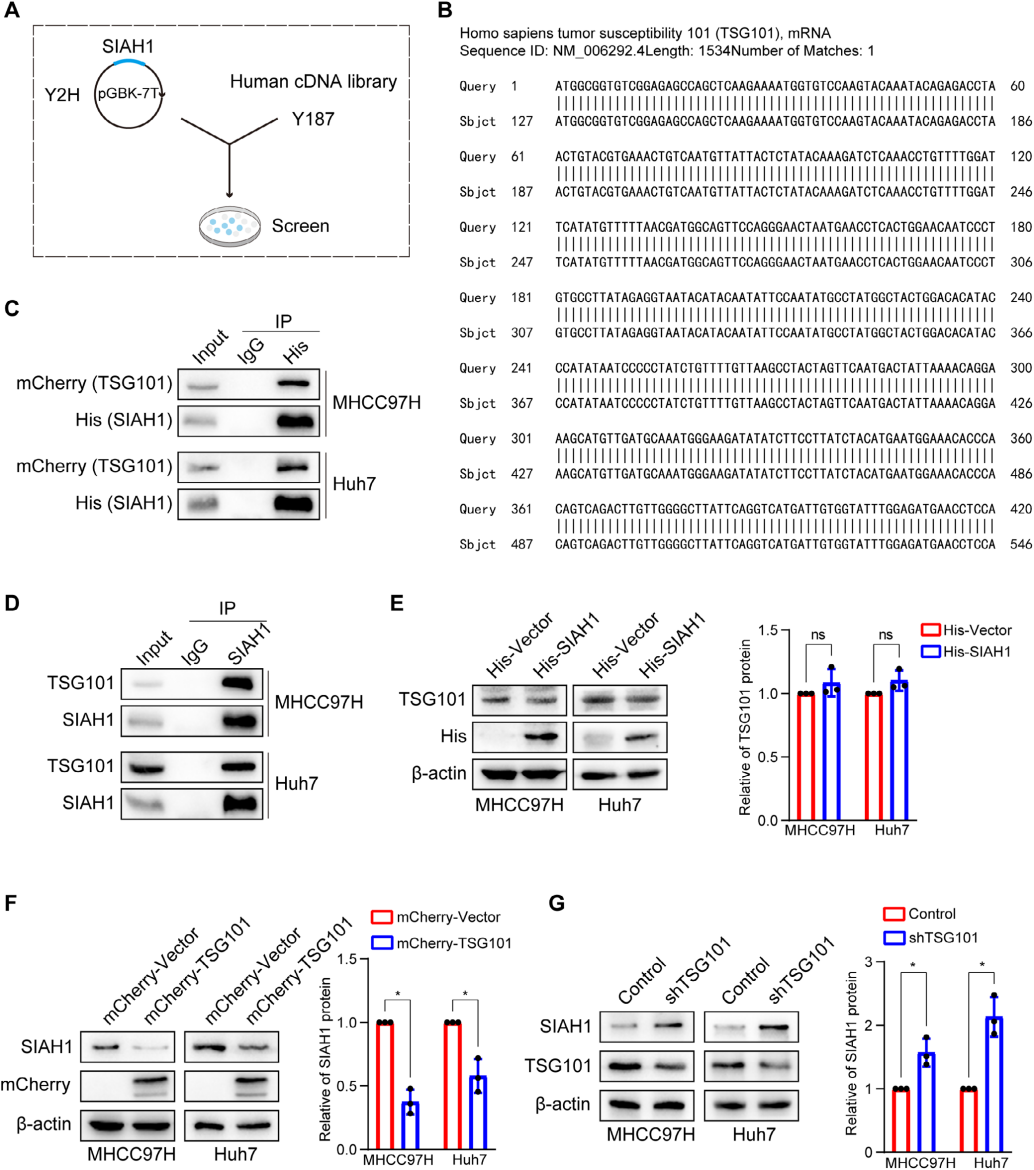
SIAH1 interacts with TSG101 in HCC cells and was down‐regulated by TSG101. (A) Yeast two‐hybrid experiment was conducted with SIAH1 bait. (B) The obtained sequence was blasted with NCBI to obtain TSG101. (C) The His‐SIAH1 and mCherry‐TSG101 plasmids were co‐transfected in MHCC97H and Huh7 cells, and His antibody was used for IP. (D) Antibody against SIAH1 was used for IP to detect the interaction between SIAH1 and TSG101. (E) The His‐Vector or His‐SIAH1 plasmids were transfected in MHCC97H and Huh7 cells, and western blotting assay was used to detect the protein levels of TSG101. (F) The mCherry‐Vector or mCherry‐TSG101 plasmids were transfected in MHCC97H and Huh7 cells, and western blotting assay was used to detect the protein levels of SIAH1. (G) Western blotting assay was used to detect the protein levels of SIAH1 and TSG101 in the stable cell lines silencing TSG101. *p < 0.05.

To analyze the reason SIAH1 is down‐regulated in HCC, a yeast two‐hybrid system was used to identify potential SIAH1‐interacting proteins. TSG101 was identified as a potential SIAH1‐interacting protein by blasting the yeast two‐hybrid results in the NCBI database (Figure 1A,B). Subsequently, co‐immunoprecipitation was performed to confirm the interaction between SIAH1 and TSG101 in MHCC97H and Huh7 cells. We co‐transfected His‐SIAH1 and mCherry‐TSG101 into HCC cells. It was found that His‐tagged SIAH1 interacted with mCherry‐tagged TSG101 in HCC cells (Figure 1C). In addition, we also found that the endogenous SIAH1 could interact with TSG101 in HCC cells (Figure 1D). These results indicated that SIAH1 interacts with TSG101 in HCC cells.

Next, we analyzed the regulatory mechanism between SIAH1 and TSG101 by transiently transfecting His‐SIAH1 or mCherry‐TSG101 in HCC cells, respectively. The results showed that overexpression of TSG101 had a stable and remarkable effect on decreasing SIAH1 protein level, while His‐SIAH1 could not affect the expression of TSG101 (Figure 1E,F). To further define the regulatory role of TSG101 on SIAH1 protein expression, shRNA target was designed to knock down TSG101 according to our previous study [23]. It was found that the protein levels of SIAH1 were increased by silencing TSG101 in HCC cells (Figure 1G). These results suggested that TSG101 is a key upstream molecule regulating SIAH1, which may be an important reason for the low expression of SIAH1 in HCC.

### 3.2 TSG101 Is Up‐Regulated in HCC and Associated With Poor Prognosis

**FIGURE 2:**
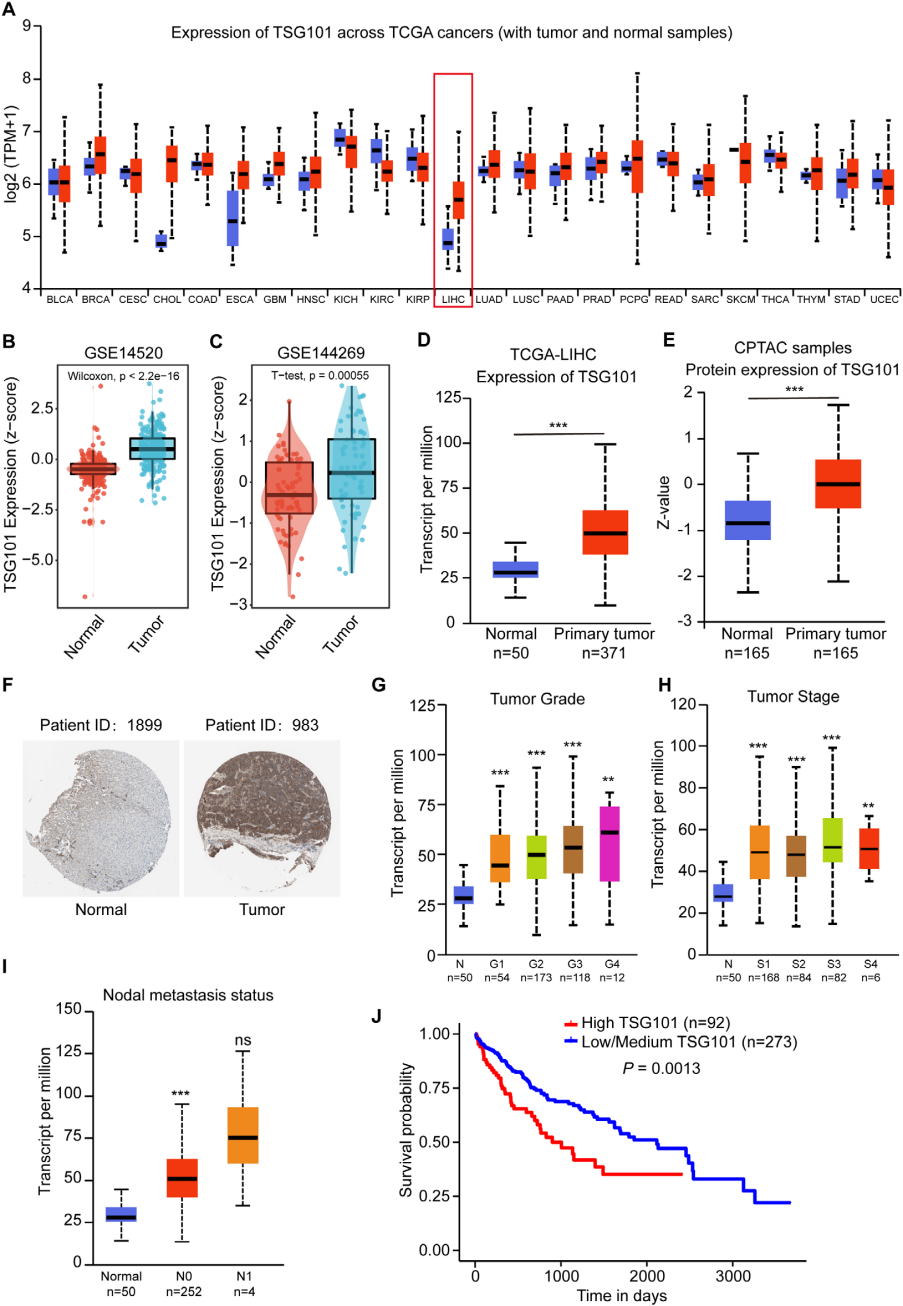
TSG101 is up‐regulated in HCC and associated with poor prognosis. (A) Pan‐cancer analysis for TSG101 expression. (B, C) TSG101 expression in GSE14520 and GSE144269. (D) TSG101 expression from TCGA‐LIHC. (E) TSG101 protein expression from CPTAC samples. (F) TSG101 expression from the Human Protein Atlas database (https://www.proteinatlas.org/). (G) Expression of TSG101 in LIHC based on tumour grade. Grade1‐Well differentiated (low grade), Grade2‐Moderately differentiated (intermediate grade), Grade3‐Poorly differentiated (high grade), Grade4‐Undifferentiated (high grade). Statistical significance: Normal vs. Grade1: 4.18090007059391E‐11; Normal vs. Grade2: < 1E‐12; Normal vs. Grade3: 1.62436730732907E‐12; Normal vs. Grade4: 3.023100E‐03. (H) Expression of TSG101 in LIHC based on cancer stages. Statistical significance: Normal vs. Stage1: 1.62447832963153E‐12; Normal vs. Stage2: 1.63224989080391E‐12; Normal vs. Stage3: 1.62447832963153E‐12; Normal vs. Stage4: 9.942300E‐03. (I) Expression of TSG101 in LIHC based on nodal metastasis status. N0: No regional lymph node metastasis; N1: Metastases in 1 to 3 axillary lymph nodes. Statistical significance: Normal‐vs‐N0: 1.62436730732907E‐12; Normal‐vs‐N1: 8.384400E‐02; N0‐vs‐N1: 2.835200E‐01. (J) Effect of TSG101 expression level on LIHC patient survival. **p, ***p < 0.001.

To clarify the important role of TSG101 in liver cancer, we first performed some bioinformatics analysis. Pan‐cancer analysis indicated that TSG101 was differentially expressed in many cancers, including liver cancer (Figure 2A). Besides, in GSE14520 and GSE144269 databases, TSG101 was also up‐regulated in tumour (Figure 2B,C). We also used UALCAN analysis platform to analyze the expression of TSG101 [25]. We found that TSG101 was up‐regulated in tumour both at mRNA levels and protein levels (Figure 2D,E). IHC also showed TSG101 had high expression in tumour tissues from The Human Protein Atlas (Figure 2F). Next, the expression of TSG101 was associated with tumour grade, stage, nodal metastasis, and patient survival (Figure 2G–J). These results indicated that TSG101 could have significant roles in the development of HCC.

### 3.3 TSG101 Promotes the Invasion and Migration of HCC Cells Through Reducing SIAH1 Protein Levels

**FIGURE 3:**
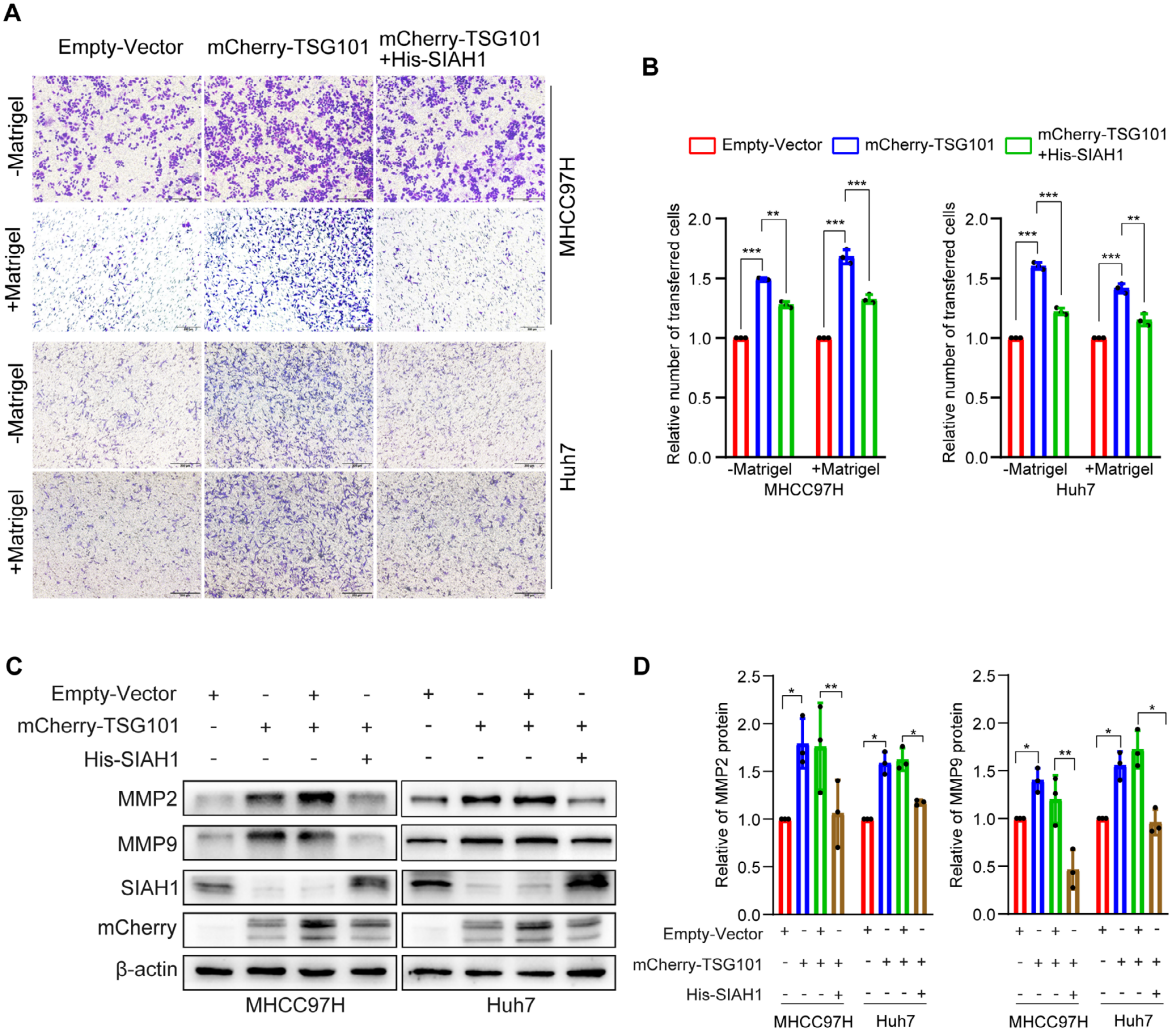
TSG101 promotes the invasion and migration of HCC cells through reducing SIAH1 protein level. (A, B) Representative transwell images and quantification for migration (−Matrigel) and invasion (+Matrigel) of MHCC97H and Huh7 cells transfected with Empty‐Vector, mCherry‐TSG101, and mCherry‐TSG101+ His‐SIAH1, scale bar: 200 μm. (C, D) Western blotting assay was used to detect the protein levels of MMP2 and MMP9 in MHCC97H and Huh7 cells transfected with Empty‐Vector, mCherry‐TSG101, mCherry‐TSG101+ His‐Vector, and mCherry‐TSG101+ His‐SIAH1. *p < 0.05, **p < 0.01, ***p < 0.001.

Then, we conducted transwell assays to detect the effects of TSG101 on the invasion and migration of HCC cells. Overexpression of TSG101 promotes the invasion and migration of HCC cells, while increasing SIAH1 expression could abate the promotional effect (Figure 3A,B). Our previous study has confirmed that TSG101 promotes the migration and invasion of HCC cells through regulating the MMP2/9 pathway [23]. Next, we further analyzed the expression of MMP2/9 through transfecting His‐SIAH1 in HCC cells overexpressing TSG101. It was found that overexpression of SIAH1 restored the protein levels of MMP2/9 induced by TSG101 up‐regulation (Figure 3C,D). The above findings indicate that TSG101 facilitates the invasion and migration of HCC cells via downregulating the protein levels of SIAH1.

### 3.4 TSG101 Decreases the Protein Stability of SIAH1 by Inducing Its Auto‐Ubiquitination

**FIGURE 4:**
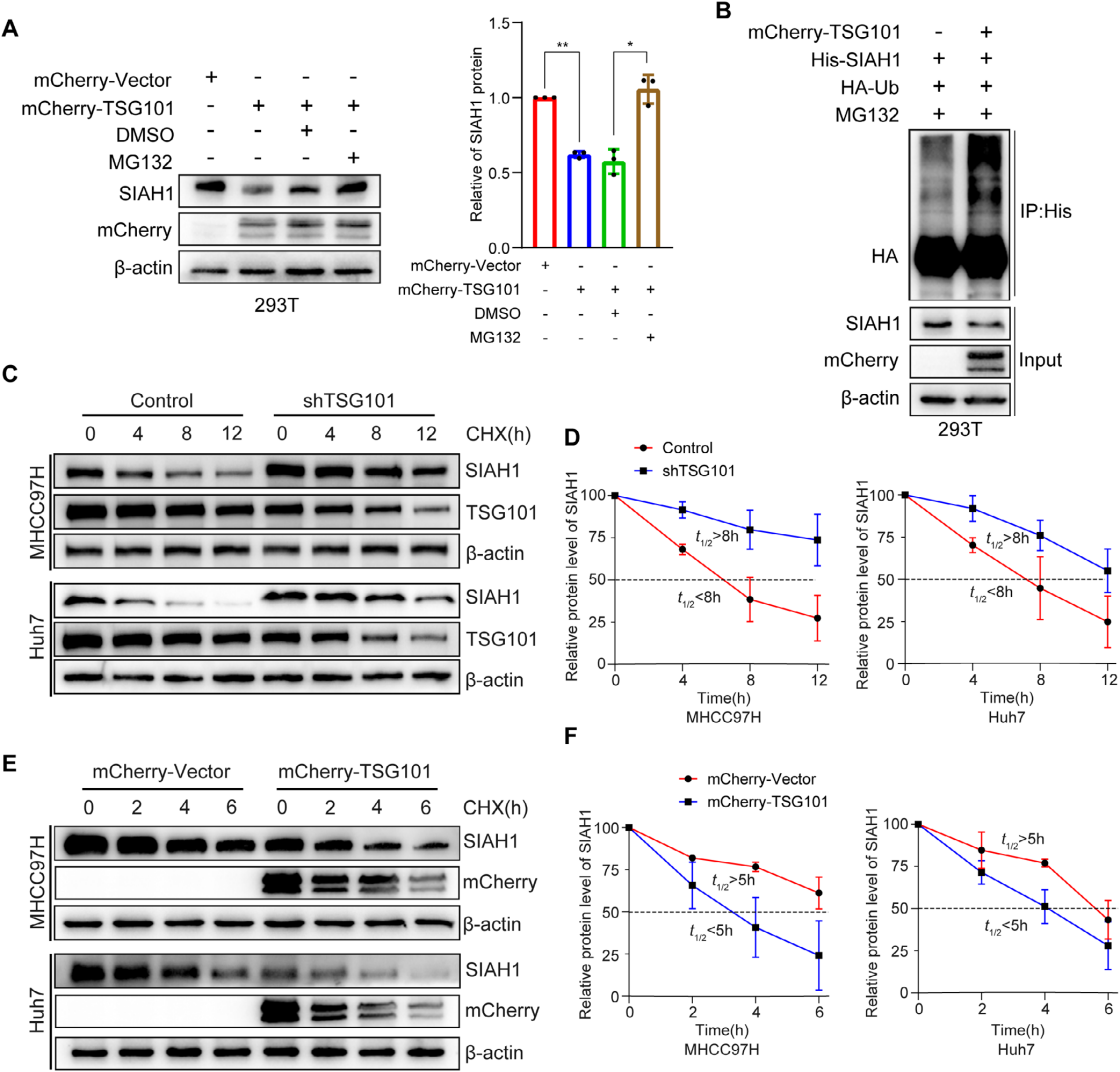
TSG101 reduces SIAH1 protein level by proteasome system. (A) Western blotting assay was used to detect the protein levels of SIAH1 in 293 T cells transfected with mCherry‐Vector and mCherry‐TSG101, and treated with DMSO or MG132. (B) The ubiquitination assay was performed to detect the ubiquitination of SIAH1.293 T cells were transfected with the mCherry‐Vector, mCherry‐TSG101, His‐SIAH1, and HA‐Ub plasmids, and treated with MG132. Antibody against His was used for IP. (C, D) Western blotting assay was used to detect the protein levels of SIAH1 in MHCC97H and Huh7 cells silencing TSG101, and treated with CHX. (E, F) The mCherry‐Vector and mCherry‐TSG101 plasmids were transfected in MHCC97H and Huh7 cells, and treated with CHX. Western blotting assay was used to detect the protein levels of SIAH1.*p < 0.05, **p < 0.01.

The ubiquitin proteasome system is one of the important pathways to modulate protein abundance in cells. It has been reported that TSG101 is a regulatory protein involved in the ubiquitination process, acting in the form of a dominant negative mutant of E2 ubiquitin binding enzyme [16]. To further explore the underlying mechanism of TSG101 regulation on SIAH1, the proteasome inhibitor (MG132) was used. It is MG132 that reinstated the protein levels of SIAH1 reduced by TSG101 (Figure 4A). This indicates that TSG101 reduces SIAH1 protein levels via the proteasome. Next, we found that overexpression of TSG101 led to an elevation in the ubiquitination levels of SIAH1 (Figure 4B). Further, we examined the effect of TSG101 on the stability of SIAH1 protein by CHX (cycloheximide) tracking assay. The overexpression of TSG101 could decrease the stability of SIAH1, while silencing TSG101 had the opposite effect (Figure 4C–F).

**FIGURE 5:**
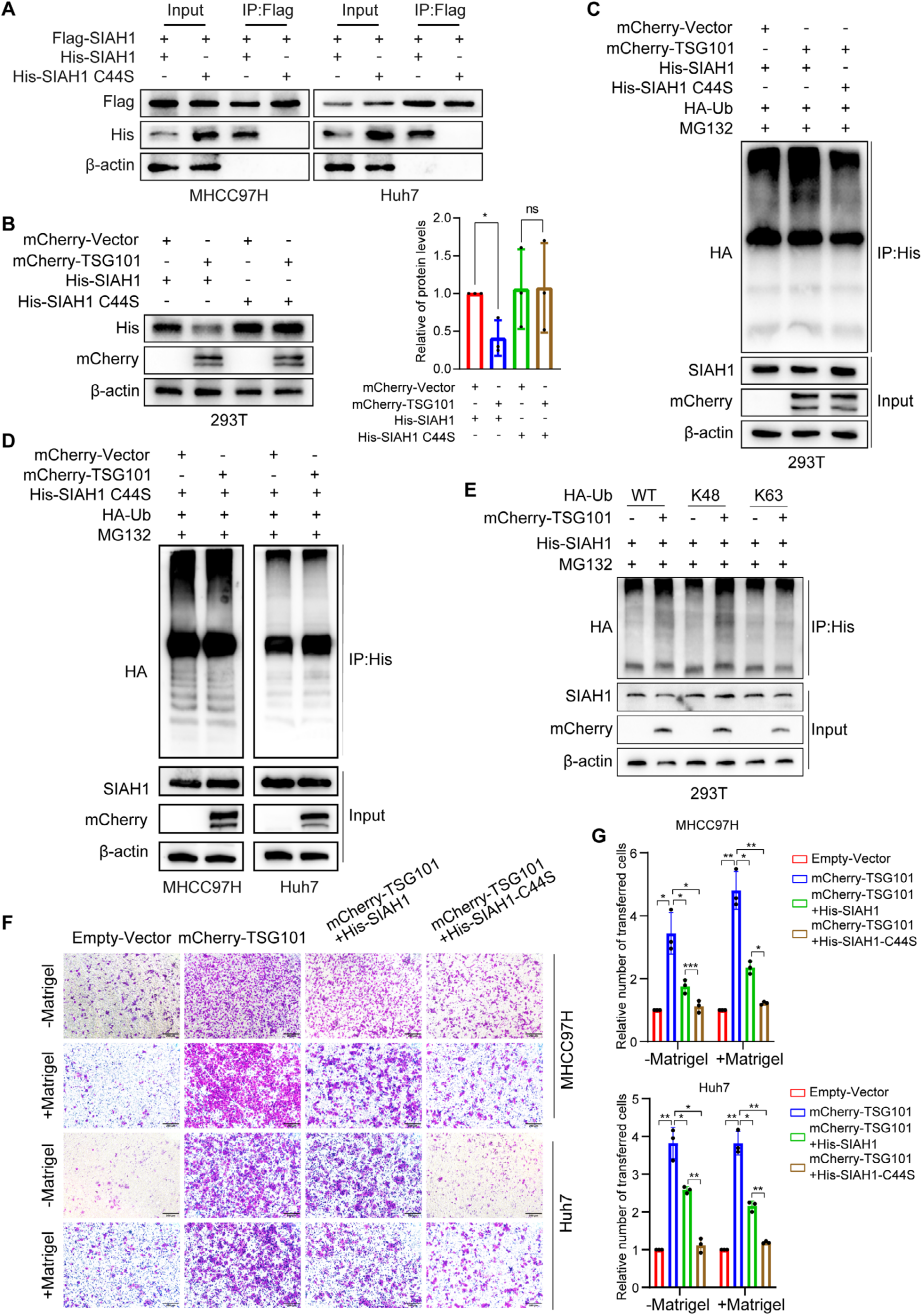
TSG101 induces the auto‐ubiquitination of SIAH1. (A) The Flag‐SIAH1, His‐SIAH1 or His‐SIAH1 C44S plasmids were transfected in MHCC97H and Huh7 cells. Antibody against Flag was used for IP to detect whether SIAH1 interacted with SIAH1 C44S. (B) The mCherry‐Vector, mCherry‐TSG101, His‐SIAH1, and His‐SIAH1 C44S were transfected, and the protein levels of SIAH1 or SIAH1 C44S were detected by western blotting. (C) The mCherry‐Vector, mCherry‐TSG101, His‐SIAH1, His‐SIAH1 C44S, and HA‐Ub were transfected, and antibody against His was used for IP. The ubiquitination level of SIAH1 or SIAH1 C44S was detected. (D) The mCherry‐Vector, mCherry‐TSG101, His‐SIAH1 C44S, and HA‐Ub were transfected, and antibody against His was used for IP. The ubiquitination level of SIAH1 C44S was detected. (E) The mCherry‐Vector, mCherry‐TSG101, His‐SIAH1, and HA‐Ub (WT, K48, K63) were transfected, and antibody against His was used for IP. The ubiquitination level of SIAH1 was detected. (F, G) Representative transwell images and quantification for migration (−Matrigel) and invasion (+Matrigel) of MHCC97H and Huh7 cells transfected with Empty‐Vector, mCherry‐TSG101, mCherry‐TSG101+ His‐SIAH1, and mCherry‐TSG101+ His‐SIAH1‐C44S. scale bar: 200 μm. *p < 0.05, **p < 0.01.

We further investigated whether SIAH1 ubiquitinates itself dependent on its E3 ligase activity. A SIAH1‐RING mutant in which Cys44 in the RING domain was converted to Serine was generated, which has been reported to be a catalytically inactive E3 [12]. We found that the SIAH1 C44S mutant lost the ability to interact with wild‐type SIAH1 (Figure 5A). Moreover, TSG101 reduced the protein levels of SIAH1, but not the SIAH1 C44S mutant (Figure 5B). We also observed that TSG101 increased the ubiquitination of SIAH1, while the ubiquitination levels of the SIAH1 C44S mutant were much weaker (Figure 5C). Besides, TSG101 could not promote the ubiquitination of SIAH1‐C44S (Figure 5D). Next, we transfected Ub‐mutant plasmids (K48 and K63); the results showed that TSG101 mainly increased the K48‐linked ubiquitination of SIAH1 (Figure 5E). In conclusion, TSG101 decreases the protein stability of SIAH1 by promoting its auto‐ubiquitination.

Next, to identify the role of the auto‐ubiquitination of SIAH1 in cell invasion and migration, we performed a rescue experiment. We overexpressed SIAH1‐WT and SIAH1‐C44S in cells with TSG101 overexpression. We found the mutant SIAH1 exhibits a stronger inhibitory effect compared to the wild‐type SIAH1 (Figure 5F,G). We believe this is because the SIAH1‐C44S doesn't undergo auto‐ubiquitination, leading to its stabilization and consequently a stronger inhibitory effect.

### 3.5 TSG101 Protein Negatively Associated With SIAH1 Protein in HCC Tissues

**FIGURE 6:**
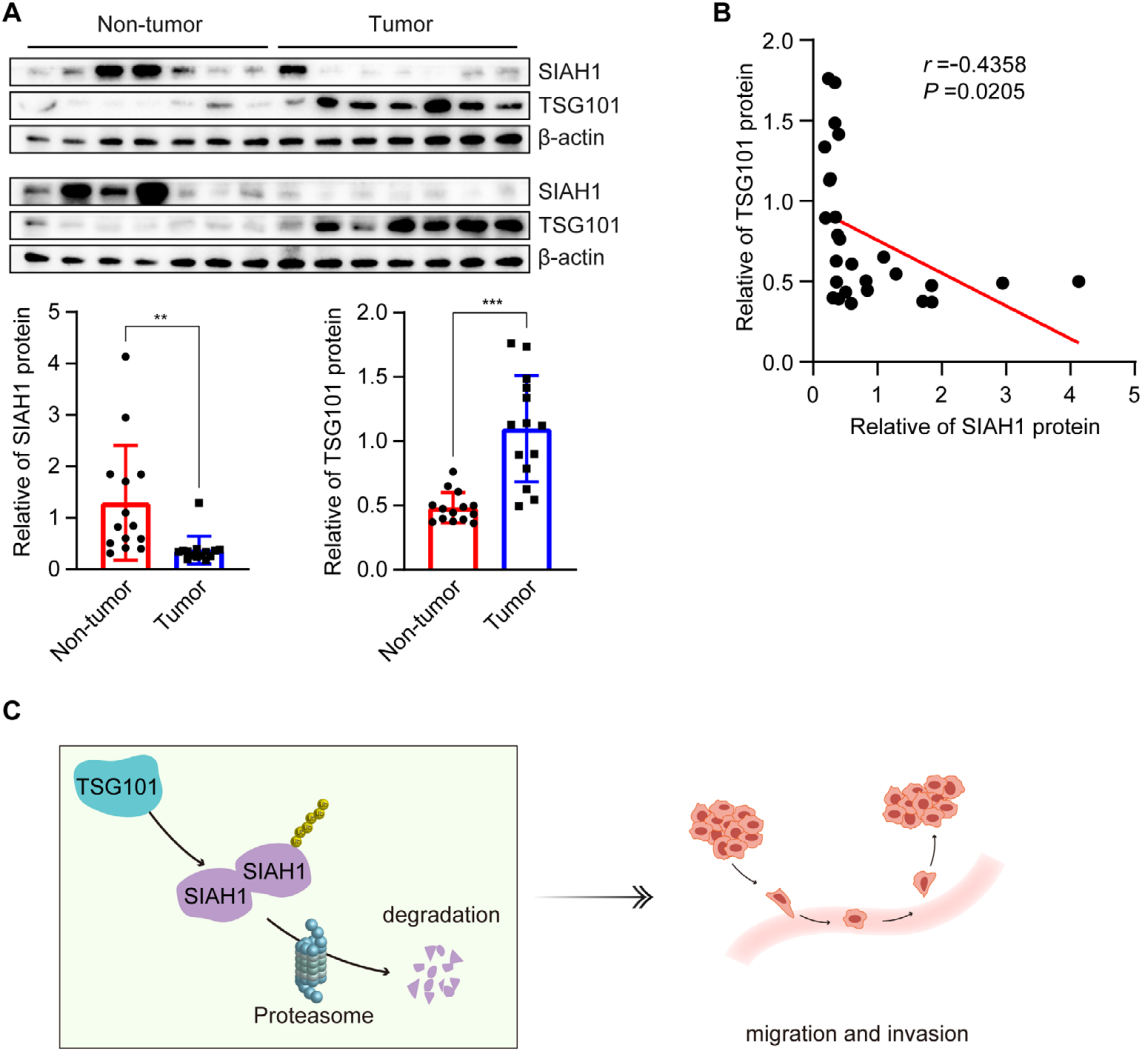
TSG101 protein negatively associated with SIAH1 protein in HCC tissues. (A) Protein levels of SIAH1 and TSG101 in HCC tissues (*n* = 14) and normal liver tissues (*n* = 14). (B) The correlation of SIAH1 expression with TSG101 in normal liver tissues and HCC tissues (r = −0.4358). (C) Schematic illustration of this study. **p < 0.01, ***p < 0.001.

Based on the above results, we identified that TSG101 induced SIAH1 auto‐ubiquitination to reduce the protein levels of SIAH1. Finally, we explored the clinical relevance of TSG101 and SIAH1. We tested the protein levels of TSG101 and SIAH1 in human HCC tissues and normal liver tissues. It was found that the protein levels of SIAH1 were significantly lower in HCC tissues than in normal liver tissues, and TSG101 was up‐regulated in HCC tissues (Figure 6A). Further, we identified a negative correlation (*r* = −0.4358) between SIAH1 and TSG101 (Figure 6B). Although the negative correlation coefficient is not strong, this may be due to the small sample size of liver cancer or the large sample heterogeneity. To some extent, *r* < −0.3 still reflects the negative correlation between TSG101 and SIAH1 in HCC tissues. In summary, TSG101 protein is negatively associated with SIAH1 protein in HCC tissues.

## 4 Discussion

The SIAH family comprises mammalian proteins similar to the Sina protein in Drosophila, and they also belong to the E3 ubiquitin ligase family [26]. It has been reported that SIAH1 is involved in a series of cell activities such as DNA damage response, hypoxia adaptation, apoptosis, angiogenesis, and cell proliferation [7, 27]. Studies have shown that SIAH1 is closely related to the occurrence and development of a variety of human cancers. For example, SIAH1 promotes the proliferation of non‐small cell lung cancer cells by ubiquitinating and stabilizing the transmembrane receptor protein Notch1 [28]. Our previous data also indicated that SIAH1 is down‐regulated in HCC and promotes the polyubiquitination and degradation of CTR9, thereby suppressing the metastasis of HCC cells [12]. Meanwhile, we have also verified that NEDD4 acts as a ubiquitin ligase for SIAH1 polyubiquitination and degradation [29]. In this study, we used yeast two‐hybrid experiments to screen proteins interacting with SIAH1 and found that TSG101 was a potential SIAH1‐interacting protein. Further co‐immunoprecipitation was used to confirm that SIAH1 interacted with SIAH1 in HCC cells.

Recent studies have found that TSG101 plays an important role in the occurrence and development of various malignant tumours. For example, TSG101 regulates the expression level of androgen receptor AR through the endosomal/lysosomal degradation pathway, thus affecting the occurrence and development of prostate cancer [30]; TSG101 promotes the proliferation, migration, and invasion of HCC cells by regulating the expression of p53, p21, and MMPs signalling pathways [23]. We have also determined that TSG101 is up‐regulated in human HCC patients, which may accelerate the proliferation, migration, and invasion of HCC cells through regulating PEG10 [23]. These suggest that TSG101 may be a potential tumour biomarker. In this study, we found that TSG101 promoted the migration and invasion of HCC cells by negatively regulating the expression of SIAH1, and this effect was related to the MMP2/MMP9 pathway. Unlike previous studies, this research primarily focuses on the regulation of SIAH1. A study has reported that SIAH1 could interact with PEG10 in HCC [31], but the regulatory mechanism is still unclear, and the relationship between SIAH1 and PEG10 will be further explored in our next study.

Studies have shown that most E3 ubiquitin ligases have their own ubiquitination activity [32]. Through auto‐ubiquitination, these E3 ubiquitin ligases play an important role in cell life activities. For example, mitochondrial damage induces the auto‐ubiquitination of E3 ubiquitin ligase TRIM5α, thus promoting mitochondrial autophagy [33]. The auto‐ubiquitination of E3 ubiquitin ligase is also closely related to the occurrence and development of tumours. For instance, CSN6 inhibits FBXW7β‐mediated ubiquitination degradation of FASN by enhancing the auto‐ubiquitination of FBXW7β, thereby positively regulating lipogenesis and promoting the development of colorectal cancer [34]; AKR1C3 regulates NF‐κB activity by regulating TRAF6 and inducing its auto‐ubiquitination in HCC cells, thereby increasing the proliferation and invasion of tumour cells [35]; PARG stabilizes c‐Myc protein in HCC cells by promoting the auto‐ubiquitination of DDB1, thereby promoting the growth and metastasis of HCC [36]. In this study, we focused on the regulatory mechanisms of SIAH1, and we found that TSG101 promoted the auto‐ubiquitination and degradation of SIAH1 through the proteasome pathway, thereby reducing its protein stability. What's more, we analyzed the protein levels of TSG101 and SIAH1 in clinical human tumour samples and found that TSG101 negatively regulated the expression of SIAH1.

## 5 Conclusion

In conclusion, this study proved that TSG101 promotes the migration and invasion of HCC cells by negatively regulating the expression of SIAH1. Further studies showed that TSG101 promoted the auto‐ubiquitination and degradation of SIAH1 through the proteasome pathway, thereby reducing its protein stability (Figure 6C). Our study reported a novel regulatory mechanism of SIAH1 and provided a new perspective for subsequent research on SIAH1. These findings provide new ideas for further research on the role of TSG101 and SIAH1 in the occurrence and development of HCC and their potential molecular mechanisms. However, whether there are other ways for TSG101 to regulate SIAH1 ubiquitination needs further study.

## Author Contributions

**Wengang Shan:** conceptualization (lead), formal analysis (equal), investigation (supporting), methodology (equal), supervision (equal), visualization (equal), writing – original draft (lead). **Xixi Gu:** conceptualization (equal), supervision (equal), visualization (equal), writing – original draft (equal). **Jinzhao Wu:** formal analysis (equal), investigation (equal), methodology (equal). **Chenrui Xu:** formal analysis (supporting), investigation (supporting), methodology (supporting). **Kai Wu:** funding acquisition (equal), project administration (supporting), supervision (supporting). **Hengliang Shi:** funding acquisition (supporting), project administration (equal), supervision (equal), writing – review and editing (lead). **Jie Qiu:** conceptualization (equal), data curation (equal), formal analysis (equal), methodology (equal), supervision (equal), visualization (equal), writing – review and editing (equal).

## Funding

This work was supported by the Postgraduate Research & Practice Innovation Program of Jiangsu Province [KYCX25_3278] and Open Project of Key Laboratories of Jiangsu Province Universities [XZSYSKF2025040].

## Ethics Statement

This study was approved by the Ethics Review Committee of the Affiliated Hospital of Xuzhou Medical University (approval no.: XYFY2019‐KL129‐01). Informed consent was obtained from all patients before treatment. The study was conducted in accordance with the Declaration of Helsinki.

## Consent

Written informed consent for publication was obtained from all participants.

## Conflicts of Interest

The authors declare no conflicts of interest.

## Data Availability

Declaration of competing interestThe authors declare that they have no known competing financial interests or personal relationships that could have appeared to influence the work reported in this paper.

## References

[1] C. Xia , X. Dong , H. Li , et al., “Cancer Statistics in China and United States, 2022: Profiles, Trends, and Determinants,” Chinese Medical Journal 135, no. 5 (2022): 584–590. 35143424 10.1097/CM9.0000000000002108 PMC8920425

[2] D. Anwanwan , S. K. Singh , S. Singh , V. Saikam , and R. Singh , “Challenges in Liver Cancer and Possible Treatment Approaches,” Biochimica Et Biophysica Acta. Reviews on Cancer 1873, no. 1 (2020): 188314. 31682895 10.1016/j.bbcan.2019.188314 PMC6981221

[3] J. M. Llovet , F. Castet , M. Heikenwalder , et al., “Immunotherapies for Hepatocellular Carcinoma,” Nature Reviews. Clinical Oncology 19, no. 3 (2022): 151–172. 10.1038/s41571-021-00573-2 34764464

[4] S. Confalonieri , M. Quarto , G. Goisis , et al., “Alterations of Ubiquitin Ligases in Human Cancer and Their Association With the Natural History of the Tumor,” Oncogene 28, no. 33 (2009): 2959–2968. 19543318 10.1038/onc.2009.156

[5] A. Weerawardhana , T. U. B. Herath , W. A. Gayan Chathuranga , et al., “SIAH1 modulates antiviral immune responses by targeting deubiquitinase USP19,” Journal of Medical Virology 96, no. 3 (2024): e29523. 38483060 10.1002/jmv.29523

[6] W. Gao , L. Chen , L. Lin , et al., “SIAH1 Reverses Chemoresistance in Epithelial Ovarian Cancer via Ubiquitination of YBX‐1,” Oncogene 11, no. 1 (2022): 13. 10.1038/s41389-022-00387-6 PMC8913663 35273154

[7] J. Wu , Y. Xue , X. Gao , and Q. Zhou , “Host Cell Factors Stimulate HIV‐1 Transcription by Antagonizing Substrate‐Binding Function of Siah1 Ubiquitin Ligase to Stabilize Transcription Elongation Factor ELL2,” Nucleic Acids Research 48, no. 13 (2020): 7321–7332. 32479599 10.1093/nar/gkaa461 PMC7367184

[8] H. R. Ko , E. J. Jin , S. B. Lee , et al., “SIAH1 ubiquitin ligase mediates ubiquitination and degradation of Akt3 in neural development,” Journal of Biological Chemistry 294, no. 42 (2019): 15435–15445. 31471318 10.1074/jbc.RA119.009618 PMC6802513

[9] M. G. Adam , S. Matt , S. Christian , et al., “SIAH Ubiquitin Ligases Regulate Breast Cancer Cell Migration and Invasion Independent of the Oxygen Status,” Cell Cycle 14, no. 23 (2015): 3734–3747. 26654769 10.1080/15384101.2015.1104441 PMC4825722

[10] C. J. Kim , Y. G. Cho , C. H. Park , et al., “Inactivating Mutations of the Siah‐1 Gene in Gastric Cancer,” Oncogene 23, no. 53 (2004): 8591–8596. 15467739 10.1038/sj.onc.1208113

[11] Z. Xiao , Z. Wei , D. Deng , et al., “Downregulation of Siah1 Promotes Colorectal Cancer Cell Proliferation and Migration by Regulating AKT and YAP Ubiquitylation and Proteasome Degradation,” Cancer Cell International 20 (2020): 50. 32082080 10.1186/s12935-020-1124-3 PMC7020597

[12] Z. Liu , P. Luo , K. Cao , et al., “SIAH1/CTR9 Axis Promotes the Epithelial‐Mesenchymal Transition of Hepatocellular Carcinoma,” Carcinogenesis 44, no. 4 (2023): 304–316. 37038329 10.1093/carcin/bgad021

[13] Z. Liu , Q. Hu , K. Cao , et al., “Deficiency of SIAH1 Promotes the Formation of Filopodia by Increasing the Accumulation of FASN in Liver Cancer,” Cell Death & Disease 15, no. 7 (2024): 537. 39075049 10.1038/s41419-024-06929-7 PMC11286965

[14] R. S. Ranaweera and X. Yang , “Auto‐Ubiquitination of Mdm2 Enhances Its Substrate Ubiquitin Ligase Activity,” Journal of Biological Chemistry 288, no. 26 (2013): 18939–18946. 23671280 10.1074/jbc.M113.454470 PMC3696669

[15] W. Xie , S. Jin , Y. Wu , et al., “Auto‐Ubiquitination of NEDD4‐1 Recruits USP13 to Facilitate Autophagy Through Deubiquitinating VPS34,” Cell Reports 30, no. 8 (2020): 2807–2819.e4. 32101753 10.1016/j.celrep.2020.01.088

[16] W. I. Sundquist , H. L. Schubert , B. N. Kelly , G. C. Hill , J. M. Holton , and C. P. Hill , “Ubiquitin Recognition by the Human TSG101 Protein,” Molecular Cell 13, no. 6 (2004): 783–789. 15053872 10.1016/s1097-2765(04)00129-7

[17] Y. S. Lin , Y. J. Chen , S. N. Cohen , and T. H. Cheng , “Identification of TSG101 Functional Domains and p21 Loci Required for TSG101‐Mediated p21 Gene Regulation,” PLoS One 8, no. 11 (2013): e79674. 24244542 10.1371/journal.pone.0079674 PMC3823576

[18] M. J. Carstens , A. Krempler , A. A. Triplett , M. van Lohuizen , and K. U. Wagner , “Cell Cycle Arrest and Cell Death Are Controlled by p53‐Dependent and p53‐Independent Mechanisms in Tsg101‐Deficient Cells,” Journal of Biological Chemistry 279, no. 34 (2004): 35984–35994. 15210712 10.1074/jbc.M400408200 PMC1201394

[19] Z. Shao , W. Ji , A. Liu , et al., “TSG101 Silencing Suppresses Hepatocellular Carcinoma Cell Growth by Inducing Cell Cycle Arrest and Autophagic Cell Death,” Medical Science Monitor 21 (2015): 3371–3379. 26537625 10.12659/MSM.894447 PMC4654595

[20] K. B. Oh , M. J. Stanton , W. W. West , G. L. Todd , and K. U. Wagner , “Tsg101 Is Upregulated in a Subset of Invasive Human Breast Cancers and Its Targeted Overexpression in Transgenic Mice Reveals Weak Oncogenic Properties for Mammary Cancer Initiation,” Oncogene 26, no. 40 (2007): 5950–5959. 17369844 10.1038/sj.onc.1210401

[21] F. Liu , Y. Yu , Y. Jin , and S. Fu , “TSG101, Identified by Screening a Cancer cDNA Library and Soft Agar Assay, Promotes Cell Proliferation in Human Lung Cancer,” Molecular Biology Reports 37, no. 6 (2010): 2829–2838. 19787439 10.1007/s11033-009-9835-5

[22] R. T. Liu , C. C. Huang , H. L. You , et al., “Overexpression of Tumor Susceptibility Gene TSG101 in Human Papillary Thyroid Carcinomas,” Oncogene 21, no. 31 (2002): 4830–4837. 12101421 10.1038/sj.onc.1205612

[23] Z. Liu , Z. Tian , K. Cao , et al., “TSG101 Promotes the Proliferation, Migration and Invasion of Hepatocellular Carcinoma Cells by Regulating the PEG10,” Journal of Cellular and Molecular Medicine 23, no. 1 (2019): 70–82. 30450735 10.1111/jcmm.13878 PMC6307771

[24] B. Zhang , Z. Y. Liu , R. Wu , et al., “Transcriptional Regulator CTR9 Promotes Hepatocellular Carcinoma Progression and Metastasis via Increasing PEG10 Transcriptional Activity,” Acta Pharmacologica Sinica 43, no. 8 (2022): 2109–2118. 34876700 10.1038/s41401-021-00812-3 PMC9343652

[25] D. S. Chandrashekar , S. K. Karthikeyan , P. K. Korla , et al., “UALCAN: An Update to the Integrated Cancer Data Analysis Platform,” Neoplasia (New York, N.Y.) 25 (2022): 18–27. 35078134 10.1016/j.neo.2022.01.001 PMC8788199

[26] C. S. F. Wong and A. Möller , “Siah: A Promising Anticancer Target,” Cancer Research 73, no. 8 (2013): 2400–2406. 23455005 10.1158/0008-5472.CAN-12-4348

[27] S. Lim , H. Y. Cho , D. G. Kim , et al., “Targeting the Interaction of AIMP2‐DX2 With HSP70 Suppresses Cancer Development,” Nature Chemical Biology 16, no. 1 (2020): 31–41. 31792442 10.1038/s41589-019-0415-2

[28] Y. Liu , Q. Li , L. Geng , et al., “Siah1 Promotes the Proliferation of NSCLC Cells Through Ubiquitinating and Stabilizing Notch1,” Experimental Cell Research 419, no. 1 (2022): 113305. 35961388 10.1016/j.yexcr.2022.113305

[29] C. Zhang , Z. Liu , X. Wang , et al., “Cathepsin K Promotes the Proliferation of Hepatocellular Carcinoma Cells Through Induction of SIAH1 Ubiquitination and Degradation,” iScience 26, no. 6 (2023): 106852. 37250786 10.1016/j.isci.2023.106852 PMC10209540

[30] Y.‐M. Lin , P.‐H. Chu , and P. Ouyang , “TSG101 Interacts With the Androgen Receptor and Attenuates Its Expression Through the Endosome/Lysosome Pathway,” Biochemical and Biophysical Research Communications 503, no. 1 (2018): 157–164. 29859188 10.1016/j.bbrc.2018.05.203

[31] H. Okabe , S. Satoh , Y. Furukawa , et al., “Involvement of PEG10 in Human Hepatocellular Carcinogenesis Through Interaction With SIAH1,” Cancer Research 63, no. 12 (2003): 3043–3048. 12810624

[32] M. Canning , C. Boutell , J. Parkinson , and R. D. Everett , “A RING Finger Ubiquitin Ligase Is Protected From Autocatalyzed Ubiquitination and Degradation by Binding to Ubiquitin‐Specific Protease USP7,” Journal of Biological Chemistry 279, no. 37 (2004): 38160–38168. 15247261 10.1074/jbc.M402885200

[33] B. Saha , H. Olsvik , G. L. Williams , et al., “TBK1 Is Ubiquitinated by TRIM5α to Assemble Mitophagy Machinery,” Cell Reports 43, no. 6 (2024): 114294. 38814780 10.1016/j.celrep.2024.114294 PMC11216866

[34] W. Wei , B. Qin , W. Wen , et al., “FBXW7β Loss‐Of‐Function Enhances FASN‐Mediated Lipogenesis and Promotes Colorectal Cancer Growth,” Signal Transduction and Targeted Therapy 8, no. 1 (2023): 187. 37202390 10.1038/s41392-023-01405-8 PMC10195794

[35] Q. Zhou , W. Tian , Z. Jiang , et al., “A Positive Feedback Loop of AKR1C3‐Mediated Activation of NF‐κB and STAT3 Facilitates Proliferation and Metastasis in Hepatocellular Carcinoma,” Cancer Research 81, no. 5 (2021): 1361–1374. 33361392 10.1158/0008-5472.CAN-20-2480

[36] M. Yu , Z. Chen , Q. Zhou , et al., “PARG Inhibition Limits HCC Progression and Potentiates the Efficacy of Immune Checkpoint Therapy,” Journal of Hepatology 77, no. 1 (2022): 140–151. 35157958 10.1016/j.jhep.2022.01.026

